# General practitioners’ knowledge of leg ulcer treatment in primary healthcare: an interview study

**DOI:** 10.1017/S1463423620000274

**Published:** 2020-09-24

**Authors:** Anne Friman, Desiree Wiegleb Edström, Britt Ebbeskog, Samuel Edelbring

**Affiliations:** 1 School of Education, Health and Social Studies, Dalarna University, Falun, Sweden; 2 School of Medical Sciences, Örebro University; 3 Affiliated to Dermatology Unit, Department of Medicine Solna, Karolinska Institutet, Sweden; 4 Department of Neurobiology, Care Sciences and Society, Karolinska Institutet, Sweden; 5 School of Health Sciences, Örebro University, Sweden

**Keywords:** experiences, GP, knowledge, knowledge development, leg ulcer treatment

## Abstract

**Aim:**

To describe general practitioners’ (GPs’) knowledge and the development of their knowledge regarding leg ulcer treatment when treating patients with leg ulceration at primary healthcare centers.

**Background:**

Earlier research regarding GPs’ knowledge of leg ulcer treatment in a primary healthcare context has focused primarily on the assessment of wounds and knowledge of wound care products. Less is known about GPs’ understandings of their own knowledge and knowledge development regarding leg ulceration in the everyday clinical context. This study, therefore, sets out to highlight these aspects from the GPs’ perspective.

**Methods:**

Semi-structured interviews were conducted with 16 individual GPs working at both private and county council run healthcare centers. The data were analyzed inductively using a thematic analysis.

**Results:**

Four themes were identified. ‘Education and training’ describe the GPs’ views regarding their knowledge and knowledge development in relation to leg ulcer treatment. ‘Experience’ refers to GPs’ thoughts about the importance of clinical experience when treating leg ulcers. ‘Prioritization’ describes the issues GPs raised around managing the different knowledge areas in their clinical work. ‘Time constraints’ explore the relationship between GPs’ sense of time pressure and their opportunities to participate in professional development courses.

**Conclusions:**

The study shows that the GPs working in primary healthcare are aware of the need for ongoing competence development concerning leg ulceration. They describe their current knowledge of leg ulcer treatment as insufficient and point to the lack of relevant courses that are adapted for their level of knowledge and the limited opportunities for clinical training.

## Background

Leg ulceration is a chronic and persistent ailment, take years to heal and frequently recur (O´Meara *et al*., 2012). For each patient, the burden of illness caused by leg ulcers is heavy, because leg ulcers typically weep, smell and reduce mobility, thus making them both incapacitating and socially isolating (Green *et al*., 2014; Hellström *et al*., 2016). Furthermore, the treatment of chronic leg ulcers can incur high costs for healthcare services, and there is often considerable pressure to implement effective care management programs (Phillips *et al*., 2015; Guest *et al*., 2017). Managing the treatment of chronic leg ulcers is, thus, a continually evolving process, with frequent introductions of new evidence/research that routinely lead to the development of new treatment methods and products (SBU, 2014; Münter, 2016; Rosenbaum *et. al*., 2018). Given that the elderly are at a much greater risk of developing an active leg ulcer, as the population ages, the number of patients requiring treatment is likely to increase (SBU, 2014). It is, therefore, worrying that several studies identify deficiencies in the management of leg ulceration in general practice (Sadler *et al*., 2006; Templeton and Telford, 2010; Gray *et al*., 2019). Patients are being treated incorrectly or are being treated without a diagnosis (Weller and Evans, 2012; Sinha and Sreedharan, 2014; Mooij and Huisman, 2016, Gray *et al*., 2018).

General practitioners (GPs) are important actors in leg ulcer care. However, previous research has shown that many GPs do not fully understand leg ulcers nor do they have the knowledge to manage leg ulcers effectively. They often lack product knowledge (Tauveron *et al*., 2004; Evans *et al*., 2010) and the ability to assess wounds accurately so as to ensure an accurate diagnosis (McGuckin and Kerstein, 1998; Graham *et al*., 2003; Sadler *et al*., 2006). Additional research has shown that GPs’ approaches to leg ulcer treatment differ significantly from those outlined in current guidelines. For example, few of them carry out ultrasound assessments, and there is a general lack of knowledge about compression therapy as an effective treatment for venous leg ulcers (Graham *et al*., 2003; Sadler *et al*., 2006; Ashby *et al*., 2014).

Considering both the clinical and financial importance of leg ulcer care, it is vital that treatment is based on current best practice and evidence-based knowledge (Nelson and Bell-Syer, 2014; Lindholm and Searle, 2016; Öien *et al*., 2016).

In Sweden, most leg ulcer treatment is performed in primary care. Current guidelines place the responsibility for the diagnosis of leg ulcers on GPs (SFAM, 2016; VISS, 2016) while nurses are responsible for dressing application, compression bandaging and patient education (Lindholm and Searle, 2016; VISS, 2016). There are major regional variations in the care and treatment of leg ulcers, and the quality of care is determined by education, competence, experience and local traditions (SBU, 2014). Despite the fact that leg ulcer treatment is an important area in Swedish primary healthcare, little is known about GPs experience in this field in the primary healthcare setting. Most of the previous research on GPs’ knowledge of leg ulceration has taken a quantitative approach (e.g., McGuckin and Kerstein, 1998; Graham *et al*., 2003). Very few studies have used a descriptive design based on GP experiences. This study, therefore, aims to describe GPs’ experiences of their knowledge and knowledge development regarding leg ulcer treatment when treating patients with leg ulceration at primary healthcare centers.

## Research methods

### Study design

In order to gain an understanding of GPs’ experiences regarding leg ulcer treatment in their clinical practice, this study used a qualitative, descriptive design with an inductive approach (Patton, 2012). Individual interviews were chosen, because GPs usually meet patients as individual health care professionals.

### Context

In Sweden, physicians who have specialized in general medicine (GPs) are the main source of medical expertise within primary care and, as a result, are the foundation upon which medical care is provided (Swartling, 2006). One of the aims of general medical training is that the GPs should get a broad and comprehensive knowledge of the biology, epidemiology and pathophysiology of the skin and be able to independently manage leg ulcers and other vascular diseases. This responsibility includes the diagnosis and treatment of leg ulcers and, when required, the referral of more serious cases to a specialist (SFAM, 2016). Courses on leg ulcer treatment are offered by Academic primary health care center once GPs enter regular practice (Academic Primary Health Care Centre, 2018).

### Data collection

Participating GPs were selected through a convenience sample keeping age, gender and experience in mind. Unit managers were asked to identify GPs who had registered patients with chronic leg ulcers. These individuals were then contacted and invited to take part in the study. A total of 16 GPs agreed to take part, all of them based at primary healthcare centers. The sample consisted of six men and ten women aged between 39 and 65 years (median = 49). At the time of the study, they had been working as registered physicians for between ten and 37 years (median = 17.5) and as specialists in general practice from one to 31 years (median = 8). One of the participants was also specialized in geriatrics. Qualitative individual interviews were carried out at the GP’s practice, which included both private and county council run healthcare centers. The interviews were based on a semi-structured interview guide with questions concerning knowledge and knowledge development in the treatment of leg ulcers. Previous quantitative research (Graham *et al*., 2003; Tauveron *et al*., 2004) describing GPs’ personal experiences in the treatment of leg ulcers was used to develop the interview guide. Interviews, thus, addressed the areas of knowledge and knowledge development, opportunities for knowledge development and factors that affect knowledge development (Table 1). Answers were followed up with more specific questions. All authors participated in data collection except BE, who reviewed the interview guide. The interviews lasted approximately 30 minutes and were recorded and transcribed verbatim. Some of this collected data has been analyzed in an earlier study by Friman *et al*. (2018).

**Table 1.** Interview guide

Area of questioning	Main question
Knowledge/knowledge development regarding the treatment of leg ulcers	How would you describe your knowledge regarding the treatment of leg ulcers? Examples of follow-up questions: If your knowledge is good →what do you think you are particularly good at? Which factors do you consider to be knowledge-intensive? What are you not so good at? Can you describe a situation that has been important for your knowledge development regarding leg ulcers?
Opportunities for knowledge development regarding the treatment of leg ulcers	What opportunities do you have for updating your knowledge regarding leg ulcers? Examples of follow-up questions: What do you do practically to update yourself about leg ulcers? What do you think is important in order to give patients a good level of care?
Factors making knowledge development regarding leg ulcer treatment difficult	Can you describe factors that make knowledge development regarding leg ulcers difficult? What are your thoughts about this?

### Data analysis

Interview data were analyzed using the method described by Braun and Clarke (2006). The analysis was divided into several steps. Each transcribed interview was first read several times in order to become acquainted with the material. The data were then organized by coding the text into meaningful elements. The next step was to identify themes by grouping related codes together. Preliminary themes were then discussed and adjusted until agreement was reached on the final themes that would be used to illuminate the purpose of the study. All authors were involved in the initial phase of the analysis while one of us [SE] was responsible for critical review of the final interpretations of the themes. An illustration of the analysis process is presented in Table 2.

**Table 2.** Illustration of the analytical steps

Data extracts	Initial coding	Interpretation	Themes
‘…very little time is given to many of the chronic diagnoses during medical school…’ (GP 7)	Little time during medical education	Knowledge of chronic wounds are not first concern during medical education	Education and training
‘I get a thousand invitations to educational courses but a course should always be chosen based on what is required most and there may be five different areas that need to be prioritized…’ (GP 14)	Prioritizing based on need	How one prioritizes determines knowledge development in wound treatment	Prioritization

### Ethical issues

The local ethics committee approved the study (Registration number 2014/615-31/1). Prior to the interviews, participants were informed about the purpose of the study and the fact that participation was voluntary and that they could withdraw at any time without consequences. Written informed consent was obtained from all participants.

## Results

The analysis resulted in four themes: ‘Education and training,’ ‘Experience,’ ‘Prioritization’ and ‘Time constraints.’ The themes are presented here and illustrated with citations from the various interviews. The numbering of the GPs in the citations indicates the order in which the interviews took place.

### Education and training

In general, the GPs highlighted the importance of their initial medical education, mainly because it had provided them with their essential professional knowledge about leg ulcer treatment. They reported that leg ulcer care had not been an area that was prioritized during their medical education. A two-hour lecture on leg ulcers was all that one of the GPs mentioned having received. That some chronic conditions such as leg ulcers were not given more attention during their training was seen by some as a disadvantage for their knowledge development regarding leg ulcer treatment. One of the newly examined GPs, reflecting on her basic undergraduate education, said:

‘I think that so little time is given to many of the chronic diagnoses during medical school and that there is a lot of focus on the bigger, grander and acute … so I think more emphasis could be given to this [leg ulcer treatment] during medical school actually, so that medical students already have better knowledge.’ (GP 7)

The GPs interviewed perceived the treatment of leg ulcers as an area requiring considerable resources. They talked about how they needed to keep up-to-date with treatment methods, which they felt changed frequently. Even though operating at a GP level, they still felt their knowledge was insufficient. They felt they had the knowledge levels to be able to accurately assess uncomplicated, even acute wounds, but not those associated with medical comorbidities. Leg ulcer care was an area that physicians tended to neglect, thus making them difficult to assess. As one GP said:

‘Usual, uncomplicated acute wounds or lacerations do not present a problem but when it comes to, in particular, stasis dermatitis, the development of leg ulcers or protracted leg ulcers then we do not have any background knowledge whatsoever. No, very poor knowledge I am afraid.’ (GP 4)

Several of the GPs pointed out that there were few opportunities for additional education and training in leg ulcer care. Of those courses that were available most were not adapted to meet the knowledge needs of physicians:

‘I do not think that as a physician, I am even eligible to take those courses or even if there are any such courses … I think … in other words if there are any aimed at physicians. It was really good when I started here, because we had a district nurse who was responsible for this area …’ (GP 10)

However, inadequate ongoing training in leg ulcer treatment seemed to be caused by its low priority and placement within the district nurses’ area of responsibility, rather than the lack of adequate courses per se.

### Experience

The GPs placed great value on the experience they acquired in their day-to-day clinical work, and this experience was considered to be the basis for improving their knowledge about leg ulcer treatment. In the case of leg ulcers, however, there was a general perception among the GPs that they rarely met patients with this condition. They felt they would need to see many more patients in order to increase their knowledge in this area. By encountering different treatment situations, they thought they would be able to build a knowledge base for the treatment of leg ulcers. Experience was considered important, for example, for the assessment of wound infection. The importance of clinical experience was addressed when the use of antibiotics was taken up as routine in the treatment of chronic leg ulcers:

‘Sometimes a colleague treats a leg ulcer with the usual Penicillin V, if that does not work, then we have to use an antibiotic with a broader effect, and then, it is Flucloxacillin that is the standard.’ (GP 14)

The GPs pointed out that, because they were not routinely involved in leg ulcer care, it was difficult for them to gain enough experience to provide a basis for knowledge development.

‘I think that I am starting to get a bit more of an idea about the venous and arterial leg ulcers, you know, from looking at the pigmentation, swelling, edema, but I still think that it is difficult, especially when it is mixed venous and arterial insufficiency, it is difficult I think, but I will gladly see them and learn more … I think that through seeing more patients, I will build up a basis and then I can read up on the rest …’ (GP 11)

The GPs made comparisons between themselves and their knowledge development with physicians in, for example, dermatology clinics, who had more frequent opportunities to gain an expertise in leg ulcer treatment. They thought that this was often a question of resources at the healthcare centers:

‘As we are just called in when there is a problem, sometimes the decisions are made quickly … you maybe would like to have more time to look through things or sit down and have a discussion, follow up the patient for a period with the nurse or so. It is a question of resources …’ (GP 13)

### Prioritization

The GPs described their work in primary care as multi-faceted in nature and requiring a broad area of knowledge. They felt that they rarely had time to gain an in-depth knowledge of any single condition, such as wounds and leg ulcer treatment. The GPs felt, however, that they ought to learn more about the treatment of leg ulcers and improve their knowledge, because it was perceived to be part of their area of responsibility. The problem for GPs was then prioritizing leg ulcers among all of the many other areas they needed to develop. As one GP commented:

‘I get a thousand invitations to educational courses but a course should always be chosen based on what is required most, and there may be five different areas that need to be prioritized…’ (GP 14)

The GPs also made a distinction between their knowledge and that which district nurses had. While they stated that they felt proficient in the etiological causes of leg ulcers, knowledge about topical treatments and holistic treatment approaches was considered to be the responsibility of the district nurses, because they take care of leg ulcers more directly:

‘As I think the nurses take quite a lot of responsibility, and some have specialist training in leg ulcer treatment, I pass a lot over to them. They keep tabs on the patient and all on that side of things.’ (GP 15)

The GPs felt that knowledge of leg ulcer treatment was a question of how their work at the healthcare centers was organized and structured. GPs each had their own area of special interest in which they had developed a more extensive knowledge. However, these specialisms did not include leg ulcer care, because it was considered to be the district nurses’ area of work. They felt that one of the district nurses could function as a resource for the whole team, keeping up-to-date with the latest developments and disseminating knowledge to the other professionals when relevant, including themselves. In general, the GPs in this study considered leg ulcer treatment to be a relatively simple and low status treatment, which did not require significant intervention on their part. They felt that their time should be devoted to more complicated conditions such as asthma and other chronic obstructive pulmonary diseases.

The GPs also considered their work environment to be challenging. They had many areas they needed to know about, they had limited time to develop specialist knowledge in any one area, and they were obliged to work within a system, which was geared toward moving patients out of the system quickly. Trying to learn more about leg ulcer treatments in these conditions was challenging:

‘We have a tough working environment, we have to do everything, and we have to prioritize. The hospital does what it has to do, and then, the patients are discharged quite early. There is not enough time. I feel that I do not know that area [dressings], the nurse should know that area, as has been said, and anyway they have more education and information about bandaging materials and the like.’ (GP 16)

### Time constraints

The GPs raised the general issue of lack of time, in particular with regard to participation in courses and continuing professional development. One reason why it was difficult to participate in courses was the large number of patients GPs had on their lists. Taking a course meant that work accumulated, requiring overtime to complete. Many of the GPs claimed that they did not have any time at all for training and preferred to rely on district nurses’ knowledge of wounds and leg ulcer treatment.

As it was so difficult to find the time to attend a course, many of the GPs reported that they used books to acquire knowledge about leg ulcer care, even if the books were old. The internet was also mentioned as a good way of keeping up-to-date, particularly websites that were aimed specifically at physicians. In addition, discussions with colleagues were considered of value, as were specialist referrals:

‘I try to see what others say about it, I discuss with colleagues, discuss with nurses and sometimes have contact with specialists, and referrals are sent, and then, you try to learn from the referral report …’ (GP 6)

## Discussion

This study shows that the GPs in primary healthcare describe a need for ongoing skill development. Earlier research has shown that physicians consider their knowledge to be inadequate, especially regarding the assessment of leg ulcers and knowledge about products for local treatment (Graham *et al*., 2003; Tauveron *et al*., 2004; Sadler *et al*., 2006). This study has produced similar findings. The GPs studied here acknowledged that their medical training had provided them with little in-depth knowledge of leg ulceration and its treatment. After several years in practice, many of them now relied on their memories or on the expertise of others, especially district nurses. Nevertheless, just as in other studies, we can identify a slow development of knowledge in this area (Patel and Granick, 2007; Yim *et al*., 2014). Reflecting the many pressures they were under, GPs expressed a reluctance to take part in continuing professional development courses, particularly those that they felt were directed toward district nurses. In order to improve leg ulcer treatment in primary care, educational activities involving nurses and GPs together could be introduced. Continuing development in this area is important, because the accurate assessment and correct diagnosis of leg ulcers set the basis for all subsequent treatment (Mooij and Huisman, 2016; Öien *et al*., 2016).

The GPs in this study discussed different ways of updating their knowledge about leg ulcer treatment. They indicated a desire to gain more experience through clinical practice, however, because of their limited involvement in this area of care; this is not a practical or efficient method of knowledge development for this professional group. Moreover, in order to build and consolidate knowledge, the reflection and processing of experience are crucial (Schön, 1992). The GPs acknowledged that their existing clinical knowledge had been developed and shaped through discussions with other colleagues, specialists and district nurses. They could see that these interactions were important. This is in line with earlier studies where an interprofessional method of working was seen as a way of increasing knowledge and improving communication between GPs and both district nurses and specialists (Chen *et al*., 2015; Friman *et al*., 2018). Such an approach could also improve the continuity of leg ulcer treatment (Öien *et al*., 2016). Some of the GPs regarded antibiotics as the first option for treatment when leg ulcers worsened. GPs are not always aware of the latest treatments for leg ulcers and are vulnerable to making suggestions that are out of date (Landis, 2008). First treatment options regularly change, and the complexities of wound infection require continued and monitored treatment, which highlights the need for clinical experience in the field (Öien and Forsell, 2013; Lindsay *et al*., 2017).

The GPs clearly saw the work they were expected to do within general practice as requiring a broad knowledge of many different areas and demanding prioritization of knowledge needs. They felt that wounds and leg ulcer care were of lower priority compared to other work areas and indicated that they relied on district nurses’ knowledge in the field. Hansson (2008) has highlighted the difference in status between different areas of medical work, where technical areas are ranked highest along with biomedical diagnoses. While GPs see the treatment of leg ulcers as quite straightforward, it is an etiological diagnosis, an action that lies within the GPs expertise, which is the basis for all future treatment (Mooij and Huisman, 2016). Thus, GPs need to work together with other healthcare professionals to lay the groundwork for effective treatment (Friman *et al*., 2018). One aspect of this, as Hansson (2008) suggests, is that compared to GPs, district nurses’ circumstances give them better opportunities to follow up with patients during longer and regularly scheduled visits, a view that is also shared by Templeton and Telford (2010). By participating in healthcare networks, district nurses are able to keep themselves up-to-date on the latest findings within their specialist area (DSF, 2018).

The GPs reluctance to spend more time learning about leg ulcer treatment was fuelled by their feeling of time pressure and the decisions they had to make about how best to use their time. Many of them said that, although there were courses available, they simply did not have the time to take them. In general, studies have shown that GPs are the group within the medical profession that devotes the least amount of time to professional development and collegial discussions (Ohlin, 2007). Financial cuts within Swedish primary care have been cited by GPs in previous studies as leaving a reduced workforce and fewer personnel to cope with rising demands and increasing workloads (Hansson, 2008). The GPs in the present study indicated that it was their patient-related work at the healthcare centers, which demanded all of their time, so hard decisions had to be made. Further training simply had to be put to one side. This is important to consider from an organizational perspective, because chronic wounds and wound care are very complex and often driven by systemic illness (Lindsay *et al*., 2017).

## Conclusions

The GPs acknowledged that their shortcomings regarding leg ulcer care, specifically in relation to leg ulcer treatment, were due to their basic medical education, which largely focused on other clinical areas, such as internal medicine, surgery and acute medical conditions. As it is mainly other staffs who manage the care of patients with leg ulcers, it is difficult for GPs to improve their experience in this area. The nature of GPs’ work, requiring knowledge of many different areas, means that other medical conditions are prioritized ahead of leg ulcer care. Time pressures also made it difficult for GPs to improve their knowledge regarding the treatment of leg ulcers; they frequently relied on the expertise of district nurses and specialists.

## Further research

Further studies need to be done on how to improve GPs’ knowledge of leg ulcer care. This should be part of a wider research area investigating the role of GPs and the knowledge they consider to be relevant to their professional practice. In addition, the organizational structure of the primary care system should be examined in order to provide the time and opportunity for GPs to gain regular professional development in the area of leg ulceration.
